# Level of Digitization in Dutch Hospitals and the Lengths of Stay of Patients with Colorectal Cancer

**DOI:** 10.1007/s10916-017-0734-3

**Published:** 2017-04-08

**Authors:** Rube van Poelgeest, Julia T. van Groningen, John H. Daniels, Kit C. Roes, Theo Wiggers, Michel W. Wouters, Guus Schrijvers

**Affiliations:** 1 Julius Center, Public Health, UMC Utrecht, Utrecht, Netherlands; 2 Department of Surgery, Leiden University Medical Center, Leiden, the Netherlands; 3 Dutch Institute for Clinical Auditing (DICA), Leiden, the Netherlands; 4 HIMSS Analytics, Chicago, IL USA; 5 UMC Utrecht, Utrecht, Netherlands; 6 University Medical Center Groningen, Groningen, Netherlands; 7 Netherlands Cancer Institute - Antoni van Leeuwenhoek, Amsterdam, Netherlands

**Keywords:** Hospital, Colorectal surgery, Quality assurance, Health care, EMR, Maturity model

## Abstract

A substantial amount of research has been published on the association between the use of electronic medical records (EMRs) and quality outcomes in U.S. hospitals, while limited research has focused on the Western European experience. The purpose of this study is to explore the association between the use of EMR technologies in Dutch hospitals and length of stay after colorectal cancer surgery. Two data sets were leveraged for this study; the HIMSS Analytics Electronic Medical Record Adoption Model (EMRAM^SM^) and the Dutch surgical colorectal audit (DSCA). The HIMSS Analytics EMRAM score was used to define a Dutch hospital’s electronic medical records (EMR) capabilities while the DSCA was used to profile colorectal surgery quality outcomes (specifically total length of stay (LOS) in the hospital and the LOS in ICU). A total of 73 hospitals with a valid EMRAM score and associated DSCA patients (*n* = 30.358) during the study period (2012–2014) were included in the comparative set. A multivariate regression method was used to test differences adjusted for case mix, year of surgery, surgical technique and for complications, as well as stratifying for academic affiliated hospitals and general hospitals. A significant negative association was observed to exist between the total LOS (relative median LOS 0,974, CI 95% 0.959–0,989) of patients treated in advanced EMR hospitals (high EMRAM score cohort) versus patients treated at less advanced EMR care settings, once the data was adjusted for the case mix, year of surgery and type of surgery (laparoscopy or laparotomy). Adjusting for complications in a subgroup of general hospitals (*n* = 39) yielded essentially the same results (relative median LOS 0,934, CI 95% 0,915–0,954). No consistent significant associations were found with respect to LOS on the ICU. The findings of this study suggest advanced EMR capabilities support a healthcare provider’s efforts to achieve desired quality outcomes and efficiency in Western European hospitals.

## Introduction

Implementations of potentially transformative eHealth technologies throughout the world frequently have a significant impact on national health expenditures. Such large-scale efforts and investments have been justified on the grounds that the EMR, picture archiving and communication systems (PACS), electronic prescribing (ePrescribing) and associated computerized provider (or physician) order entry systems (CPOE), and computerized decision support systems (CDSS) are supposed to help to address the problems of variable quality and safety in modern health care [1–6]. However, the scientific basis of such claims, which are repeatedly made and seemingly uncritically accepted, remains to be firmly established.

For the measurement of the level of implementation of information systems a concept of maturity of these systems has been developed. There is a large number of methods or models available to measure the level of implementation of information technology [7]. One of these methods is the so-called Electronic Medical Record Adoption Model (EMRAM) scoring approach developed by Healthcare Information and Management Systems Society (HIMSS) Analytics [8]. EMRAM is an eight stage maturation model reflecting the EMR capabilities in hospitals, ranging from a completely paper-based environment (Stage 0) to a highly advanced paperless and digital patient record environment (Stage 7). The scoring process is done by identifying the software used in the different functional areas of the hospital. At least 150 questions per hospital are included about demographics, software functionalities, processes, integration standards, usage in percentage by physician and nurses, depending on the available software in the hospital. Previous studies on this model in the Netherlands show that EMRAM stage 3, the first stage in which clinical functionalities (nursing) become available, presents as the first notable challenge to Dutch hospitals; 37.5% of the hospitals in this study have yet to satisfy the requirements of this stage. The basic and more advanced clinical capabilities should help to increase the quality, safety and efficiency of the treatment of patients in the hospital [9].

The Dutch surgical colorectal audit (DSCA), started in 2009, is a nationwide audit used to monitor, evaluate and improve quality of care of primary colorectal cancer surgery. It provides feedback to all hospitals in the Netherlands on a set of quality measures and indicators. While EMRs may lead to better hospital performance and outcomes, hospitals may use EMRs to improve the quality of care. This paper has the objective to contribute to the scientific discourse on the relationship between the digitalization of hospital data and the effect on quality of care, with colorectal cancer as a guiding example [10, 11].

Our thesis behind this study is that when basic clinical functionalities (EMRAM > =3) are available in a hospital, the patients will have a more efficient hospitalization. Efficient communication could prevent medical or organizational mistakes and make it possible to transfer patients from an intensive care unit (ICU) to a general ward and transfer patients from the ward to home without undue delay. We used the post-operative length of stay (LOS) in this study because that is where the presumed effect is expected. Preoperative patients are usually admitted to the hospital the same day or the day before the surgery.

**Fig. 1:**
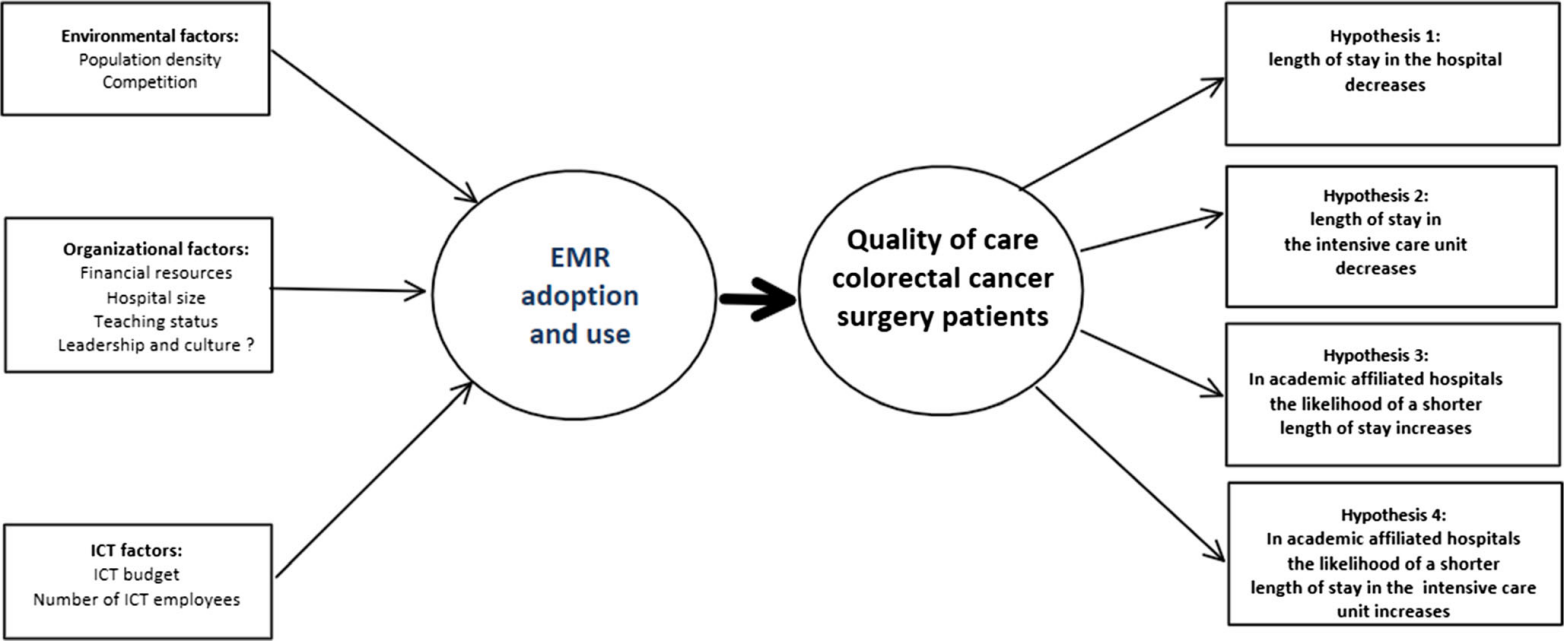
The theoretical model

University and top teaching hospitals provide a great deal of specialized care and medical research, as well as the training and education of many of the nation’s health care providers. Former studies [12, 13] indicate that academic affiliated hospitals may more easily adapt to changes than general hospitals. According to Retchin and Wenzel [13], university health centers, as well as top teaching hospitals, can easily adapt to the use of EMRs because they, “have the expertise to resolve remaining software issues, the components necessary for the integrated delivery, a culture for innovation in clinical practice, and a generation of future providers that can be acclimated to the requisites for computerized records”(p.493 of Retchin and Wenzel(13)). Another reason for this increased likelihood is that medical training occurs in these hospitals, and younger medical trainees tend to be more comfortable with computers as they have recently used them in school [14]. Because of this, the staff resistance to EMR use may not be as great as in other hospitals [13]. Based upon these properties we expect that in academic affiliated hospitals the above mentioned effect is even stronger. From this model (Fig. 1) we deducted the following hypotheses.

- *Hypothesis 1: In hospitals with more advanced EMR capabilities the likelihood of a shorter LOS on average of colorectal cancer surgery patients in the hospital increases*.
- *Hypothesis 2: In hospitals with more advanced EMR capabilities the likelihood of a shorter LOS on average in the ICU of colorectal cancer surgery patients increases.*
- *Hypothesis 3: The likelihood of a shorter LOS on average of colorectal cancer surgery patients increases in academic affiliated hospitals with more advanced EMR capabilities.*
- *Hypothesis 4: The likelihood of a shorter LOS on average in the ICU of colorectal cancer surgery patients increases in academic affiliated hospitals with more advanced EMR capabilities.*

### Methods

Data were collected from the DSCA. This disease specific registry contains information on patient, tumor, treatment and short-term outcome characteristics. All hospitals in the Netherlands register their primary colorectal cancer patients that undergo a resection in this database. Details of this dataset regarding collection and methodology have been published previously. [15, 16]

### Patients

All patients undergoing surgical resection for primary colorectal cancer between January 1, 2012, and December 31, 2014, and registered in the DSCA before March 30, 2015, were evaluated in this study. For this study no ethical approval or informed consent was required under Dutch law. Minimal data requirements to consider a patient eligible for analyses were information on tumor location, date of surgery, and mortality. Patients with local excisions were excluded (*n* = 393) Patients with LOS of 0 or less, or LOS that was missing were excluded (*n* = 358), because the origin of this outcome is possibly grounded on registration mistakes.

### Hospitals

Every hospital in the NL (*N* = 93) was invited to participate in the EMRAM study. In 2014, 73 hospitals (80%) joined the EMRAM program. Of this group of hospitals (*N* = 34) are considered academic affiliated (university and top teaching hospitals) for the purpose of this study. These hospitals provide high-complex care, lead the way in innovation and research and train (surgical) residents. For the primary objective of this study, the EMRAM scores of the hospitals present in both databases (EMRAM and DCSA) were dichotomized into hospitals with clinical functionalities (EMRAM > = 3) and hospitals without clinical functionalities (EMRAM < 3).

### Outcomes

Length of stay is shown in days, calculated by subtracting the date of surgery from the date of dismissal from the hospital. Length of stay on the ICU is registered directly into the registry.

## Statistical analysis

The primary analysis is a multivariate regression analysis on the logarithmically transformed LOS, adjusted for patient and tumor characteristics, year of surgery, hospital type and type of surgery (laparoscopy of laparotomy). Patient and tumor characteristics adjusted for are: gender, BMI-index, age, ASA classification, primary location of the tumor, pathological T stage, metastasis, perioperative tumor complications, urgency, additional resections for tumor growth and metastasis. Details concerning the use of relevant case-mix factors have been described elsewhere [16, 17]. We repeated the multivariate regression, by additionally adjusting for surgical complications in three stages: a single complication, a complication combined with reoperation and a complication leading to death. The analyses are repeated for the total group of hospitals (*N* = 73), the group of academic affiliated hospitals (*N* = 34) and the group of general hospitals (*N* = 39). Significance was considered for the primary research question, with a *p*-value < 0.05.

## Results

### Patients and hospitals

In total 73 hospitals, including 30.358 patients were included in this study. In Table 1 the distribution of patients’ characteristics among the EMRAM low group and the EMRAM high group are shown. A significant effect (relative median LOS = 0,974, CI 95% 0.959–0,989) is found between patients in the EMRAM low group and the LOS in the EMRAM high hospital group when corrected for the case mix, year of operation and type of surgery (laparoscopy or laparotomy). Additional adjustment for patients with complications confirms the association (relative median LOS 0,969, CI 95% 0,956–0,981). For LOS in the ICU the multivariate regression does not show a significant association of higher EMRAM score with smaller LOS (relative median LOS 0,995, CI 95% 0,942–1050). After adjustment for patients with complications there is also no significant association (relative median LOS 1010 CI 95% 0,962–1060) (Table 2).

**Table 1:** Patient and hospital characteristic per EMRAM Group

Patient and hospital characteristics		EMRAM-score			
		EMRAM < 3		EMRAM > =3	
		Count	Column N %	Count	Column N %
Sex	Male	4462	55,8%	9341	55,1%
	Female	3540	44,2%	7627	44,9%
BMI categories with missing	Unknown	199	2,50%	451	2,7%
	<18.5	170	2,1%	276	1,6%
	18.5–25	3195	39,9%	6758	39,8%
	25–30	3110	38,8%	6732	39,7%
	30+	1334	16,7%	2759	16,3%
Age	<=60	1528	19,1%	2972	17,5%
	61–70	2538	31,7%	5341	31,5%
	71–80	2674	33,4%	5903	34,8%
	> = 81	1268	15,8%	2747	16,2%
Charlson score in 3 groups	Charlson score 0	3967	49,5%	8586	50,6%
	Charlson score 1	1813	22,6%	3877	22,8%
	Charlson score 2+	2228	27,8%	4513	26,6%
ASA score in 3 groups	I – II	6157	77,0%	13,099	77,2%
	III	1732	21,6%	3620	21,3%
	IV – V	112	1,4%	254	1,5%
Location of tumor	Caecum	1126	14,1%	2321	13,7%
	Appendix	40	0,5%	89	0,5%
	Ascending colon	1043	13,0%	2221	13,1%
	Hepatic flexure	315	3,9%	751	4,4%
	Transverse colon	413	5,2%	1013	6,0%
	Splenic flexure	195	2,4%	403	2,4%
	Descending colon	368	4,6%	773	4,6%
	Sigmoideal colon	2307	28,8%	4617	27,2%
	Rectum	2201	27,5%	4788	28,2%
Pathological T stage	Tx/T0	23	0,3%	35	0,2%
	T1	812	10,2%	1693	10,1%
	T2	1564	19,7%	3390	20,2%
	T3	4544	57,1%	9442	56,2%
	T4	1012	12,7%	2255	13,4%
Distant metastasis	No/missing	7092	88,6%	15,190	89,5%
	Yes	916	11,4%	1786	10,5%
Pre-operative tumor complications	No/missing	5330	66,6%	9619	56,7%
	Yes	2678	33,4%	7357	43,3%
Urgent/not urgent	Elective (incl. After stent)	6864	85,8%	14,741	86,9%
	Urgent/Emergency	1140	14,2%	2227	13,1%
Additional resection because of metastasis	No	7643	95,4%	16,470	97,0%
	Yes	365	4,6%	506	3,0%
Additional resection because of extensive tumor growth	No	7209	90,0%	15,489	91,2%
	Extensive	353	4,4%	682	4,0%
	Limited	446	5,6%	805	4,7%
Surgical technique	Laparotomy	3424	43,0%	6864	40,6%
	Laparoscopy	4546	57,0%	10,039	59,4%
Complications	No complications	5570	69,8%	11,362	67,1%
	Complication	1431	17,9%	3400	20,1%
	Complications and reintervention	770	9,6%	1777	10,5%
	Complications and death	214	2,7%	403	2,4%
Size of hospital admitted	Small	1489	18,6%	2006	11,8%
	Medium	3711	46,3%	5789	34,1%
	Large	2808	35,1%	9181	54,1%
Type of hospital admitted	General hospitals	4446	55,5%	5965	35,1%
	Academic affiliated	3562	44,5%	11,011	64,9%
Region of hospital admitted	East	1271	15,9%	3438	20,3%
	North	1517	18,9%	1728	10,2%
	South	1727	21,6%	5547	32,7%
	West	3493	43,6%	6263	36,9%

**Table 2:** Length of stay of patient in total hospital group

	Univariate regression			Multivariate regression***			Multivariate regression****		
	B	95% C.I.for B		B	95% C.I.for B		B	95% C.I.for B	
		Lower	Upper		Lower	Upper		Lower	Upper
	Length of Stay (LOS) in the hospital								
Median LOS > =3/Median LOS <3	0,998	0,981	1016	0,974	0,959	0,989	0,969	0,956	0,981
	Length of Stay (LOS) in the ICU								
Median LOS > =3/Median LOS <3	1106	1047	1169	0,995	0,942	1050	1010	0,962	1060

***adjusted for: case-mix, year of registration, hospital type and technique of treatment (laparoscopic/laparotomy)

****adjusted for: as before plus complications

Looking at the subgroup of academic affiliated hospitals (*N* = 34) (Table 3) the significance of the change of the LOS in the hospital when corrected for the case mix, year of operation and type of surgery (laparoscopy or laparotomy) disappears. Still, when corrected for complications there is a significant decreasing effect.

**Table 3:** Length of stay (LOS) for patients in academic affiliated hospitals

	Univariate regression			Multivariate regression***			Multivariate regression****		
	B	95% C.I.for B		B	95% C.I.for B		B	95% C.I.for B	
		Lower	Upper		Lower	Upper		Lower	Upper
	Length of Stay (LOS) in the hospital								
Median LOS > =3/Median LOS <3	0,987	0,963	1011	0,991	0,971	1011	0,967	0,950	0,982
	Length of Stay (LOS) in the ICU								
Median LOS > =3/Median LOS <3	1093	1002	1192	1011	0,939	1089	1017	0,952	1086

***adjusted for: case-mix, year of registration, hospital type and technique of treatment (laparoscopic/laparotomy)

****adjusted for: as before plus complications

Looking at the subgroup of general hospitals (*n* = 39) (Table 4) a significant negative association (relative median LOS 0,934, CI 95% 0,915–0,954) is found when corrected for the case mix. This means an estimated decrease of the median LOS of 6,6% and 6,1% when also corrected for complications (e^B1^ = 0,939). For LOS in de ICU the multivariate regression shows a significant (relative median LOS 1104, CI 95% 1036–1177) increase for hospitals with higher EMRAM scores. When additionally adjusted for complications, there are no significant associations.

**Table 4:** Length of stay (LOS) for patients in general hospitals

	Univariate regression			Multivariate regression***			Multivariate regression****		
	B	95% C.I.for B		B	95% C.I.for B		B	95% C.I.for B	
		Lower	Upper		Lower	Upper		Lower	Upper
	Length of Stay (LOS) in the hospital								
Median LOS > =3/Median LOS <3	0,973	0,948	0,998	0,934	0,915	0,954	0,939	0,922	0,956
	Length of Stay (LOS) in the ICU								
Median LOS > =3/Median LOS <3	1077	1000	1161	1104	1036	1177	1056	0,998	1115

***adjusted for: case-mix, year of registration, hospital type and technique of treatment (laparoscopic/laparotomy)

****adjusted for: as before plus complications

## Results of tests regarding the hypotheses

Hypothesis 1 (in hospitals with more advanced EMR capabilities the likelihood of a shorter LOS on average of colorectal cancer surgery patients in the hospital increases) is supported by our findings. Hypothesis 2 (in hospitals with more advanced EMR capabilities the likelihood of a shorter LOS on average in the ICU of colorectal cancer surgery patients increases) is not supported by our findings. On the contrary a not significant increase of the LOS in the ICU is found. Hypothesis 3 (the likelihood of a shorter LOS on average of colorectal cancer surgery patients increases in academic affiliated hospitals with more advanced EMR capabilities) is not supported by our findings. On the contrary a stronger effect is measured by general hospitals instead by academic affiliated hospitals. Hypothesis 4 (the likelihood of a shorter LOS on average in the ICU of colorectal cancer surgery patients increases in academic affiliated hospitals with more advanced EMR capabilities) is also not supported by our findings.

## Discussion

For this study we tested the relation between the availability of clinical software in the hospital (EMRAM stage 3 and higher) and the LOS. For the total group of hospitals, we found a significant association as expected; LOS is shorter in hospitals with more advanced clinical software. Looking in more detail at the group of hospitals we found that the correlation is stronger in general hospitals than in academic affiliated hospitals, even when corrected for their different case mix [11]. A possible reason behind this difference might be that the academic affiliated hospitals have had EMRs longer, thus they have already made some macro adjustments that affect LOS and general hospitals are not yet as mature in EMR use and thus are still deriving the initial benefits. In addition, it is shown [18] that resident involvement may increase LOS in advanced laparoscopic surgery. This could mask the effect of the EMR.

Not clear is the slightly larger LOS in the ICU of EMRAM stage 3 or higher hospitals, especially in general hospitals (significant after case-mix correction). The difference could lie in the different levels of ICU; the least advanced ICU level is in the Netherlands frequently used for extended recovery. After repeating the analyses of general hospitals with exclusion of the lowest ICU level we see the correlation changes to a decreasing ratio, but after correction for complications also this is not significant anymore (data not shown). During our EMRAM investigation of the hospitals we found out that the software used in the ICU and the operating room, the so called Patient Data Management System (PDMS), is most of the time not integrated with the EMR system of the entire hospital. So the management of the LOS in the ICU may differ from the management of the LOS in the hospital. Only at EMRAM stage 6 and 7 the integration of the EMR system with the PDMS system is mandatory. It may also be a side effect of the diversity of hospitals in the EMRAM high group. In this group, hospitals with basic clinical facilities are present, but also hospitals with more advanced digital processes and evidence based intelligence. If in future more hospitals will reach the highest, full digital, stage 7 level a third group (EMRAM > =6) can be added to look for the association with more advanced (outcome) indicators in the DSCA database. Further research is suggested to look for this relationship.

## Limitations of the study and suggestions for further research

There are limitations to our study. First, although we achieved a 77% response rate, the hospitals that did not respond to our survey were somewhat different from those that did respond. Small hospitals and hospitals located in the northern part of the Netherlands were underrepresented in the study. The 72 hospitals that did participate provided a fairly good representation of the total population of the Netherlands 93 hospitals. Given that non responding hospitals were more likely to have characteristics associated with lower levels of adoption of electronic health records, residual bias may have led us to overestimate adoption levels. Furthermore, although we adjusted for an extensive number of patient and tumor factors, unknown confounding factors could still be present.

## Conclusion

We found a significant association between the level of digitalization of hospitals and the length of stay after colorectal cancer surgery, consistent with shorter length of stay in hospitals with higher levels of digitization.
